# Indicators of Healthcare Services Utilization among the Syrian Refugee Population in Jordan: An Observational Study

**DOI:** 10.3390/healthcare11040478

**Published:** 2023-02-07

**Authors:** Mahmoud Al-Qadi, Mahmoud Al-Hussami, Elena Riza, Esra’a Athamnah, Jumana Shehadeh, Christos Kleisiaris, Wafa Hamad Almegewly, Savvato Karavasileiadou

**Affiliations:** 1 Royal Medical Services, Amman 11855, Jordan; 2 Community Nursing Department, School of Nursing, The University of Jordan, Amman 11942, Jordan; 3 Department of Hygiene, Epidemiology and Medical Statistics, Medical School, National and Kapodistrian University of Athens, Mikras Asias 75, 11527 Athens, Greece; 4 Health Center Medical Laboratory Technician, Jordan University of Science and Technology, Irbid 22110, Jordan; 5 Department of Nursing, School of Health Sciences, Hellenic Mediterranean University, 71410 Heraklion, Greece; 6 Department of Community Health Nursing, College of Nursing, Princess Nourah bint Abdulrahman University, Riyadh 11671, Saudi Arabia

**Keywords:** healthcare access, healthcare utilization, non-communicable diseases, Syrian refugees, refugee camp

## Abstract

Background: Sufficient healthcare services utilization among the Syrian refugee population is one of the most important human rights. Vulnerable populations, such as refugees, are often deprived of sufficient access to healthcare services. Even when healthcare services are accessible, refugees vary in their level of utilization of these services and their health-seeking behavior. Purpose: This study aims to examine the status and indicators of healthcare service access and utilization among adult Syrian refugees with non-communicable diseases residing in two refugee camps. Methods: The cross-sectional descriptive design was conducted by enrolling 455 adult Syrian refugees residing in the Al-Za’atari and Azraq camps in northern Jordan, using demographical data, perceived health, and the “Access to healthcare services” module, which is a part of the Canadian Community Health Survey (CCHS). A logistic regression model with binary outcomes was used to explore the accuracy of the variables influencing the utilization of healthcare services. The individual indicators were examined further out of 14 variables, according to the Anderson model. Specifically, the model consisted of healthcare indicators and demographic variables to find out if they have any effect on healthcare services utilization. Results: Descriptive data showed that the mean age of the study participants (n = 455) was 49.45 years (SD = 10.48), and 60.2% (n = 274) were females. In addition, 63.7% (n = 290), of them were married; 50.5% (n = 230) held elementary school-level degrees; and the majority 83.3% (n = 379) were unemployed. As expected, the vast majority have no health insurance. The mean overall food security score was 13 out of 24 (±3.5). Difficulty in accessing healthcare services among Syrian refugees in Jordan’s camps was significantly predicted by gender. “Transportation problems, other than fee problems” (mean 4.25, SD = 1.11) and “Unable to afford transportation fees” (mean 4.27, SD = 1.12) were identified as the most important barriers to accessing healthcare services. Conclusion: Healthcare services must imply all possible measures to make them more affordable to refugees, particularly older, unemployed refugees with large families. High-quality fresh food and clean drinking water are needed to improve health outcomes in camps.

## 1. Introduction

Jordan is home to refugees from all over the Middle East and Africa, with nearly 800,000 registered refugees [1]. Among the refugees are 655,000 Syrians, 67,000 Iraqis, 15,000 Yemenis, 6000 Sudanese, and 2500 refugees of 52 other nationalities [1] Refugees in Jordan are also having difficulty finding work due to a variety of legal issues, forcing the majority of them to rely on non-governmental organizations for financial assistance. According to the United Nations Higher Commission for Refugees (UNHCR), refugees’ average monthly household income of 177 Jordanian dinars (JD) (249 US$) equates to just 32 JD (45 US$) per capita, well below Jordan’s poverty line of JD 68 (96 US$), and just above the country’s abject poverty line of JD 28 (40 US$), per capita per month [2]. It is reported that socioeconomic insecurity and financial concerns are linked to a high prevalence of health problems, including NCDs [3].

As stated by the World Health Organization (WHO), “the enjoyment of the highest attainable standard of health is one of the fundamental rights of every human being without distinction of race, religion and political belief, economic or social condition”. Thus, the right to health is globally recognized as a fundamental human right, without any discrimination regarding race, age, ethnicity, or any other important characteristic, among all residents of any country, including refugees. From this acknowledgment derives the legal responsibility of governments to guarantee access to timely, acceptable, and affordable healthcare of high quality, as well as to ensure the essential determinants of health, such as safe and potable water, housing, sanitation, food, health-related information and education, and gender equality [4], for the whole population of their countries.

The most important health outcome indicator is healthcare services utilization, a substitute measure of access to healthcare that alters the outcomes of health status and user satisfaction. Understanding the nature of healthcare services utilization is important for healthcare system planning, allocation, and prioritizing of resources. The WHO state [4] that all people should have access to the health services they need without the risk of financial difficulties, to facilitate acceptable access to health services without financial or other kinds of barriers [5]. While minorities’ healthcare needs are often greatly increased due to poor determinants of health, the utilization of health services by ethnic minorities is often hindered by a lack of knowledge of the healthcare system and inadequate language skills [6].

The primary goal of the healthcare service is to provide effective and appropriate care to regain or at least improve the wellbeing of the individual; it is also responsible for a range of preventive measures to protect the public from disease. According to the WHO, over 41 million deaths each year are attributed to non-communicable diseases (NCDs) alone, which constitute almost 70% of the gross mortality worldwide [7]. Noncommunicable diseases are present in all countries, regardless of their developmental level [2,3]. It has been demonstrated that certain communities within the same country showed higher morbidity from NCDs compared to the general population, and Syrian refugees are an example of this. Implementing primary healthcare through enhancing access to healthcare services, prevention and promotion of health events, proper treatment, and timely access to healthcare services is linked to improved health status, including the reduced burden of NCDs [8].

Interestingly, non-communicable diseases not only cause premature death, but also have significant negative effects on the quality of life of people affected, along with significant adverse economic impacts on individuals, communities, and societies [9]. Furthermore, there is clear evidence that, at lower costs, primary care will have greater health outcomes [10]. Thus, individuals with NCDs and those with risk indicators for NCD need proactive, patient-centered, community-based, and affordable long-term care [11].

The study’s findings on health determinants and barriers to accessing healthcare as indicators of health equity and their effect on the health of Syrian refugees in Jordan are necessary to inform policy developers, decision-makers, researchers, and other stakeholders about Syrian refugees’ needs in Jordan. This study could assist researchers and organizations who are concerned with refugees’ health in addressing social determinants of health equity and access among Syrian refugees, as well as assist them in developing and evaluating programs to enhance health equity and accessibility. Moreover, it may reduce and eliminate health disparities, which will result in improvement of Syrian refugees’ health and create equitable and accessible health systems, which in turn will also enhance their health outcomes and quality of life. To the researchers’ knowledge, only a few studies have examined the indicators that affect healthcare services utilization among Syrian refugees, and even fewer have addressed non-communicable diseases in Jordan. It is obvious, therefore, that the investigation of the existing gap regarding the indicators of health access of Syrian refugees in Jordan, taking into consideration the barriers related to accessing healthcare and chronic diseases as reasons for encounters, is crucially important.

Hence, the purpose of this study is to examine indicators that affect healthcare services utilization among Syrian refugees with chronic medical conditions in Jordan, in addition to exploring healthcare-seeking behaviors, the barriers they face in meeting their health needs, and indicators of these difficulties.

## 2. Materials and Methods

### 2.1. Study Design

A cross-sectional descriptive design was used, where data were collected at one point in time to create a clear picture of indicators of healthcare services utilization among Syrian refugees with chronic diseases residing in refugee camps in Jordan.

### 2.2. Population and Eligibility

The target population of the study was adult Syrian refugees in Jordan’s camps with non-communicable diseases that require ongoing medical attention, with a special focus on the most prevalent chronic diseases, such as hypertension, diabetes, cardiovascular diseases, chronic respiratory diseases, and/or arthritis. In addition, refugees with access to any official public or private healthcare service points in Jordan were targeted. The accessible population was patients living in Al-Za’atari or Azraq Syrian refugee camps in northern Jordan with chronic medical conditions, which are defined broadly as diseases that last 1 year or longer and require constant medical attention, and are viewed as preventable but difficult to cure naturally. The inclusion criteria referred to any Syrian refugee, 30 years and above, who arrived in Jordan due to the Syrian conflict in 2011, suffering from one or more chronic diseases, such as diabetes, hypertension, cardiovascular diseases, chronic respiratory diseases, and arthritis. In addition, participants should be registered Syrian refugees residing inside one of the UNHCR’s official refugee camps in Jordan, namely Al-Za’atari or Azraq, accessing healthcare facilities, being able to write and read the Arabic language, and agreeing to participate voluntarily. Exclusion criteria included refugees who were disabled, handicapped, and those who received psychological counseling.

### 2.3. Sample and Sampling

A non-probability, convenience-sampling method was utilized in this study to collect data from patients who suffered from chronic diseases, due to the logistical restrictions that made it very difficult to utilize a probability sampling technique. The sample size was determined based on an estimation calculation on the software G*Power using normal approximation and using the Z statistic instead of the t statistic calculation equation, where alpha was determined at 0.05, beta at 0.20, and an intermediate estimated effect size r of 0.30. The calculation yielded a minimum required sample size of 356. An additional 25% was added to account for any possible missing data, accounting for a total sample of 445 subjects.

### 2.4. Measurement Instrument

Study variables were measured using an Arabic, quantitative, self-reported survey, and modified by the authors, comprised three sections: demographical data; perceived health; and the “Access to healthcare services” module, which is a part of the Canadian Community Health Survey (CCHS). The first part is related to socio-demographical data that was developed by authors and was consistent with the literature and framework and contained the social determinants of health (age, gender, educational level, resident area, work status, health insurance, and household monthly income). The second part is related to chronic conditions, asking about the presence of the chronic condition.

The second section of the questionnaire, the perceived health status scale, consists of three items. The first two items are 5-point Likert scales asking about how the participants perceived their physical and psychological wellbeing (excellent; very good; good; fair; bad), and the third is a 1–10 scale about the participant’s overall satisfaction of their health (1 meaning strongly dissatisfied, and 10 meaning strongly satisfied).

The third section of the questionnaire, which is adopted from the “Access to healthcare services” module and the literature, is composed of four parts: the first part is related to access and use of outpatient clinics (four items); the second part is the “Barriers to Access Scale”, consisting of 20 items with 5-point Likert scale (never; rarely; sometimes; usually; always); the third part relates to patients’ experiences of waiting times in various healthcare services; and the last section is the “General Access Scale”, composed of 18 items. This scale contain four parts, one for the overall access to healthcare (five items), relationship between patients and physician (six items), and continuity of care (six items). The response for these parts was using five-point Likert scale (1 = never, 2 = rarely, 3 = sometimes, 4 = usually, 5 = always). The final part (one item) assess the overall patient’s satisfaction of provided services. The response for this part was using rating scale from one to ten (one means strongly dissatisfied, and ten means strongly satisfied).

The CCHS is a self-reported, cross-sectional survey related to health status, healthcare utilization, and health determinants. The CCHS is a 98% representative survey of Canadians aged 12 years and over who live in the provinces and territories of Canada. The CCHS started in 2001 and has been performed every 2 years since development, to collect additional information on health services and access to healthcare. The psychometric properties of the questionnaire are not available, so the psychometric properties (reliability and validity), were calculated in the pilot study. The original questionnaire is in the English language, so translation to the Arabic language was conducted.

To ensure the face validity of the instrument, a panel of subject-matter experts reviewed the questionnaire items and approved them. As for the reliability of the instrument, a pilot study was conducted, and satisfactory Cronbach’s alpha values of the service satisfaction and food security sections of the questionnaire were found. Values of Cronbach’s alpha of the three aforementioned sections of the questionnaire were 0.83, 0.91, and 0.88, respectively.

### 2.5. Data Collection Procedure

Data collection was conducted between January 2021 and April 2021. The authors employed two teams of data collectors: the team had two individuals, one male and one female. Teams were trained for 3 days on the methods of sampling, data collection skills, and how to administer the questionnaire. The training included a pilot test for the questionnaire.

A convenience sample of adult patients with diabetes, hypertension, cardiovascular diseases, chronic respiratory diseases and/or arthritis who have access to the outpatients’ clinic in the two camps’ primary healthcare centers were contacted personally by the data collector teams. The team introduced themselves to patients and explained the study purpose and the importance of their participation. The teams asked the patients about the history of their diseases; if participants said “yes”, questioning was continued and their history of accessing the primary healthcare centers was collected. The patients who met the inclusion criteria were asked to participate in the study. The patients who agreed to participate were approached by face-to-face data collectors who administered the questionnaire with emphasis on following general safety measures, such as wearing face masks and a physical distance of about 1.5 m from the participants during answering of the questionnaire. The completion of the questionnaires was conducted in a private nurses’ room, in order to ensure privacy for the eligible participants, after the necessary explanations regarding the nature and the purpose of the study, their right to withdraw from the study at any time, and reassurance regarding the protection of their privacy, anonymity, and confidentiality. In particular, the candidates were informed of the following: (1) no patient names or identifying information will be recorded; (2) all findings are reported in aggregate; and (3) questionnaires will be destroyed after the data are compiled for statistical analysis. Furthermore, they were assured that their participation would not affect the quality of care they are receiving. Finally, written consent forms were signed by the participants before data collection.

A total number of 700 refugees were accessed; 550 of them met inclusion and exclusion criteria; and 455 agreed to participate and have completed the questionnaire. Response rate was good, at 87.1%. The data collector team read the items and asked the participant to choose the responses that best represented their perceptions of their physical and psychological wellbeing (excellent; very good; good; fair; bad); the questionnaire required 15 min to be answered. A double-entry method was used to control inconsistencies during data entry, where someone reviewed the data entered and checked the authenticity of the entry against the original questionnaire.

### 2.6. Ethical Considerations

Ethical approval for this study was granted from the Scientific and Ethical Research Committees at the School of Nursing, University of Jordan (protocol code: 291; date of approval: 1 October 2021). An informed consent form was signed by each subject, containing a thorough description of the purpose and merit of the study, and explaining clearly that participation is voluntary and that each subject has the right to withdraw at any point, or refuse to answer any of the items without any consequences. The confidentiality of all study subjects was maintained throughout the study. The anonymity of subjects was preserved, as their names and contact information were not collected in the questionnaire.

### 2.7. Data Analysis

The analytical procedures are arranged by the sequence of the survey questions and the research questions. It includes data screening and preparation for analysis, including a detailed description of participants’ characteristics using descriptive analysis (central tendency and dispersion). Quantitative data were presented as mean ± standard deviation (SD), and range in qualitative data were presented as number (n) and percentage (%). In addition, inferential analysis was used to predict accessibility difficulties to healthcare services among Syrian refugees with chronic diseases through binary logistic regression analysis. Statistical analyses were carried out using SPSS, version 24.

## 3. Results

### 3.1. Socio-Demographic Characteristics

In the present study, 479 Syrian refugees were invited to participate; 455 of those agreed to participate and completed the questionnaire, with a response rate of 92.9%. Results indicated that 60.2% (n = 274), of participants were females, and 39.8% (n = 181) were males, with a mean age of 49.45 years (SD = 10.48). Concerning social status, 63.7% (n = 290), of them were married, 31.4% (n = 143), were widowed, 2.9% (n = 13), were divorced, and 2% (n = 9) were single. Half (50.5%, n = 230) were holding an elementary school-level degree, and the majority (83.3%, n = 379) were not working. Of these, 65.9% (n = 300) were from the Al-Za’atari camp, and 34.1% (n = 155) were from the Azraq camp. The mean monthly income was 42.37 (SD = 73.07) JD (Table 1).

### 3.2. Health Behaviour/General Health Characteristics

Table 2 shows the general health characteristics of the study participants and the five most prevalent chronic diseases according to their medical records (history). Particularly, more than half of the sample has either or both diabetes mellitus and hypertension, with 26.2% (n = 119) and 23.7% (n = 108), respectively. However, the rest of the participants suffering from cardiovascular diseases, chronic respiratory diseases and/or arthritis, (21.7%, n = 99; 15.4%, n = 70; 13%, n = 59). Moreover, only 1.1% (n = 5), have health insurance and, remarkably, only 0.4% (n = 4) reported “Not regular follow-up” for their chronic disease condition. Regarding smoking status; the prevalence of cigarette smokers is 22.4% (n = 102) and 2.6% (n = 12) are shisha smokers. Moreover, the majority of the sample “never/almost never do 1 hour and 15 min of strong exercise or 2 hours and a half of fair activity every week”, with 24.4% (n = 111); however, as low as 2% (n = 37) of them reported “almost always do exercises”. Regarding fruit portions, most of the participants (93.2%, n = 424) stated that they “sometimes” “eat five portions or more of fruits or vegetables”, and 91.2% (n = 415) of them reported that they “eat 3 portions or more pieces of bread” “every day”. Regarding different meat types, 80.7% (n = 367) “usually” “eat red meat”; 81.5% (n = 371) “sometimes” “eat chicken”; and 41.3% (n = 188) of them reported “never” “eat[ing] fish”. Additionally, for (Very unsatisfied/Unsatisfied), the percentage in terms of waiting times was 63.7% (n = 290); for (Very satisfied/satisfied), the percentage was only 1.5% (n = 7), while, for healthcare services, the (Very unsatisfied/Unsatisfied) percentage received was 40.9% (n = 186) and was 17.4% (n = 79) for (Very satisfied/satisfied) (Table 2).

### 3.3. Food Security

Eight items were used to assess the participants’ food security status. Lower scores mean less food insecurity among participants, and the overall scores ranged from 0 to 24, with a mean score of 13.06 (SD = 3.47). Using the quartile equation, the result revealed 32.7% (n = 149) of participants have a low level of food insecurity, and 22.4% (n = 102) of them have a high level of food insecurity. With the highest mean difficulty to the lowest, the greatest barrier seems to be “In the past 12 months, we have been worried that food would run out before you got money to buy more”, with a mean of 2.03 (SD = 0.62), followed by the item “In the past 12 months, the food bought just didn’t last, and there wasn’t any money to get more”, with a mean score of 2.02 (SD = 0.65), whereas the item “In the past 12 months, did (any of the children) ever not eat for a whole day because there wasn’t enough money for food” scored the least, with a mean score of 1.08 (SD = 0.36).

### 3.4. Healthcare Service Utilization

Binary logistic regression was developed using the “enter” mode to explore the indicators that affect the healthcare services utilization among Syrian refugees in the camp’s population in Jordan. The logistic regression goodness of fit indicators were assessed using the Hosmer–Lemeshow test and the Omnibus Tests of Model Coefficient (X^2^(23, 413) = 0, *p* = 1 and X^2^(33, 413) = 80.512, *p* < 0.001), respectively, and the results show that both indices are have a good fit. Moreover, the −2 likelihood for this study is 0, and the score of Nagelkerke-R² of 1 for this logistic regression model suggested that the accuracy of the variables influencing the utilization of healthcare services can be explained by the predictor variables under study being only 1%. Additionally, the ‘Classification Table’, which compares actual and predicted groups to assess how many would be correctly classified, was assessed. Results indicated that 98.7% of individuals were correctly classified using the null model (everyone was classified as non-utilizers of healthcare services,) and 100% were correctly classified using the full model, which is an improvement; the individual indicators were examined further, and out of the 14 variables identified, according to the Anderson model, none of them was a significant predictor.

### 3.5. Access to Healthcare

Results revealed that almost 98.2% (n = 447) of the participants stated that they have difficulties in getting the specialist care needed for a diagnosis or consultation; on the other hand, as low as 20.7% (n = 94) said that they have difficulties in getting necessary surgeries. Concerning where they follow-up on their chronic conditions, most of them 90.5% (n = 412) reported that they follow-up in NGO clinics, whereas 7% (n = 32) followed up in a public health center. Concerning the perceived distance to the healthcare facility, around two-thirds of them (59.6%, n = 271) perceived that the distance between their houses and the clinic is far. Regarding the type of transportation, half of them (50.3%, n = 229) reported that they walk to reach the healthcare facility. Regarding the time to reach the healthcare facility, 52.1% (n = 237), took more than 30 min to reach their healthcare facility, and only 9.4% (n = 43), took less than 15 min.

### 3.6. Barriers to Accessing Healthcare Services

Sixteen items were used to assess the participants’ accessibility difficulties, and the overall scores ranged from 0 to 80; lower scores mean fewer difficulties in accessing healthcare services, and the total mean difficulty score was 57.70 (SD = 13.53). From the highest mean difficulty to the lowest, the greatest barrier seems to be “Transportation problems, other than fee problems” with a mean of 4.25 (SD = 1.11), then the following item “Unable to afford transportation fees”, with a mean of 4.27 (SD = 1.12), whereas the item “Language Problems”, with a mean of 1.11 (SD = 0.64), and the item “Did not know where to go (i.e., information problems), or no one tells you where to go”, with a mean of 2.25 (SD = 1.09), were the smallest barriers.

### 3.7. Indicators for Accessibility Difficulties to Healthcare Services among Syrian Refugees in Camps Diseases in Jordan

The total difficulty with accessing healthcare ranged from zero to 80; lower scores mean fewer difficulties in accessing healthcare services. The actual mean score of all items in the construct was 57.70 (SD = 13.53). Using the quartile equation exposed that 27.3% (n = 124) of Syrian refugees in camps have few difficulties in accessing healthcare services, and 18.7% (n = 85) of them have many difficulties in accessing healthcare services. Standard multiple linear regression was performed. The results of the regression (Table 2) show that the model explained (R^2^ = 47.1%) of the variance, and that the model was a significant predictor of accessibility difficulties to healthcare services, F (14, 440) = 27.943, *p* < 0.000. The individual indicators were examined further and, out of the 14 variables identified according to Anderson model, only four of them were significant, which are living place (β = 0.494, *p* < 0.001); food insecurity (β = 0.082, *p* = 0.038); average distance for all clinics (β = 0.147, *p* < 0.001); and satisfaction regarding the waiting time to access healthcare services (β = −0.137, *p* = 0.003). Of the four previously significant mentioned predictors, one (satisfaction regarding the waiting time to access healthcare services) is inversely associated with the accessibility difficulties to healthcare services; this means that, once these variable increases, accessibility difficulties decrease. The other three significant indicators were positive predictors; this means that, once these variables increase, accessibility difficulties increase. Moreover, it seems that the “living place” (B = 14.106, *p* < 0.001) had the strongest positive predictive relationship to the dependent variable. This reflects that the participants in the Azraq camp have 14.106 units of accessibility difficulty, which is higher than participants in the Al-Za’atari camp. The final unstandardized predictive model was accessibility difficulties to healthcare services = 24.758 + (14.106 × living place) + (0.640 × food insecurity) + (9.117 × perceived distance to the healthcare services) + (−10.504 × satisfaction of the waiting time to get healthcare services).

### 3.8. Unmet Healthcare Needs

Analysis showed that 91% (n = 414) of Syrian refugees in camps reported they had unmet healthcare needs during the past 12 months. The reasons for unmet healthcare needs are presented; the most common reasons were “Do not have a regular healthcare provider”, with a mean of 4.30 (SD = 1.43), followed by “Believed they would receive inadequate care”, with a mean of 3.98 (SD = 1.54), and then “Transportation issues”, with a mean of 3.95 (SD = 1.46). The least common reason was “Appointment was cancelled”, with a mean of 2.17 (SD = 1.34). The total unmet health needs range from 0 to 45; lower scores mean less unmet needs, The actual mean score of all items in the construct was 22.65 (SD = 8.99).

This is a clear warning sign about rapid declines in providing healthcare services. Using the quartile equation exposed that 25.3% (n = 115) of Syrian refugees in camps have unmet needs related to inadequate healthcare services; however, 20.9% (n = 95) of the sample reported that they were suffering from poor healthcare services.

### 3.9. Indicators for the Unmet Healthcare Needs

Standard multiple linear regression was performed (Table 3); that is, all the predictor variables are entered into the analysis in one step, to investigate whether predisposing factors, enabling characteristics, and need variables could significantly predict participants’ unmet healthcare needs. The results of the regression analysis (Table 4) show that the model explained (R^2^= 53.3%) of the variance and that the model was a significant predictor of unmet healthcare needs, F (14, 440) = 38.037, *p* < 0.000. The individual indicators were examined further, and out of 14 variables identified according to Anderson model, six of them were significant, which are: gender (β = −0.086, *p* = 0.013); family size (β = 0.112, *p* = 0.002); living place (β = 0.265, *p* < 0.001); working status (β = −0.138, *p* < 0.001); self-perception of physical health (β = −0.280, *p* < 0.001); and food insecurity (β = 0.161, *p* < 0.001). Of the four previously significant mentioned predictors, three (gender; working status; self-perception of physical health) are inversely associated with unmet healthcare needs. This means that, once this variable increases, unmet healthcare need decreases. Specifically, the male gender, employee working status, and better self-perception of physical health are associated with less unmet healthcare needs. Moreover, it seems that the “self-perception of physical health” (B = −3.431, *p* < 0.001) had the strongest negative predictive relationship to the dependent variable.

The other three significant indicators (family number; living place; and food insecurity) were positive predictors; this means that once this variable increases, unmet healthcare needs increase. Specifically, families with more members, living in the Al-Azraq camp, and higher food insecurity levels are associated with more unmet healthcare needs. Moreover, it seems that the “living place” (B = 5.020, *p* < 0.001) had the strongest positive predictive relationship to the dependent variable. The final unstandardized predictive model was unmet healthcare needs = 22.580 + (−1.572 × gender), + (4.398 × family number) + (15.060 × living place) + (−6.884 × working status) + (−17.155 × self-perception of physical health) + (2.508 × food insecurity).

### 3.10. Perception of Health Status

Descriptive statistics were used to measure the three domains of the perception of health status among Syrian refugees in camps in Jordan. The three domains of perception of health status were “Self-perception of general health”, “Self-perception of mental health” and “Self-perception of stress in life”. Analysis showed that the mean response of self-perception of physical health was 2.01 (SD = 0.74), indicating that participants tend to perceive their physical health as fair. In addition, the mean response of self-perception of mental health was 1.83 (SD = 0.70), indicating that participants tend to perceive their mental health as poor. Moreover, the mean response of self-perception of stress in life was 3.29 (SD = 0.93), indicating that participants tend to perceive their lives as “A bit stressful”.

## 4. Discussion

This study examined the indicators affecting healthcare services utilization among Syrian refugees with non-communicable diseases in Jordan. Healthcare-seeking behaviors, barriers they face in meeting their health needs, and indicators of these difficulties were explored. Data showed that difficulty in accessing healthcare services among Syrian refugees in Jordan’s camps was significantly predicted by gender. In addition, the most important indicators to accessing healthcare services related to the refugee population characteristics and the higher prevalence rates of chronic diseases, including unmet healthcare needs such as prominent levels of food insecurity.

Healthcare service access was found to be reasonable in the current study, but Syrian refugees, including those who lack proper health insurance and have low socio-economic status, reported barriers. These findings are similar to those of Alduraidi and colleagues [12], who found that Syrian refugees inside camps in Jordan might suffer more than their non-camp counterparts in terms of socioeconomic status and personal social capital in general. In addition, the current study found that low educational levels are prevalent among Syrian refugee camp communities, which is consistent with a study by Rehr and colleagues [13], which found an association between negative health outcomes and low educational levels among Syrian refugees with NCDs [14].

It was also found that indicators of difficulty in healthcare service utilization include indicators related to the healthcare system and others related to the refugee population characteristics. One of the system-related indicators was long waiting times inside healthcare facilities. This finding is consistent with the findings of Al-Rousan and colleagues [15], who found that resources, including human resources, in healthcare facilities are very limited, causing longer waiting times in the facility [16]. The current study found a link between poorly designed healthcare services, programs, and dissatisfaction among Syrian refugee patients. This finding is consistent with Maconick and colleagues’ qualitative study, which found that healthcare programs provided to Syrian refugees should consider re-designing their services to be culturally relevant, to maximize utilization of these important healthcare services [17].

Importantly, more than half of the Syrian refugees in the sample suffered from one or both of the two main chronic diseases, such as diabetes mellitus and hypertension, while the rest of the participants suffered from cardiovascular diseases, chronic respiratory diseases, and/or arthritis. This finding is consistent with the literature, which was remarkably similar to the findings of Reher and colleagues’ study, where 44.7% of refugees reported having one or both of these two chronic diseases [13]. In addition, chronic medical conditions among Syrian refugees seem to be more prevalent compared to Jordan’s general population, as more than half of Syrian refugee households in Jordan report a member with chronic disease [16]. The UNHCR works in partnership with Jordan’s Ministry of Health to provide the necessary healthcare services to Syrian refugees both inside and outside camps in Jordan. In terms of lifestyle, the current study found that very few Syrian refugees exercise regularly and that most eat bread several times daily. These findings are consistent with the findings of Ratnayake and colleagues [18], who observed a high rate of people being overweight, which was associated with poor health outcomes among Syrian refugees with chronic illnesses, namely diabetes and hypertension. Furthermore, cigarette- and shisha-smoking were prevalent among over a quarter of the current study’s sample, according to Doocy and his colleagues [14].

As far as unemployment is concerned, the current study revealed a seriously high rate of lack of employment among Syrian refugees inside camps, which is consistent with a study by Alduraidi and Waters [19], which found a concentration of poverty and unemployment among refugee populations in general, and within refugee camps in particular. Additionally, the current study showed poorer healthcare service utilization among female refugees with chronic illnesses, which can also be linked to the fact that unemployment rates are often higher among female refugees. This is due to several cultural reasons, including different family roles in the Arab culture between men, who are expected to ensure sufficient income for the family, and women, who usually are expected to take care of the domestic responsibilities at home.

In terms of refugee-related factors, the current study found that refugees who belong to larger families and are poorly educated and unemployed are more likely to face difficulties in accessing and utilizing healthcare services. These findings are consistent with those of McNatt and colleagues [20], who stressed the importance of the cost-effectiveness of healthcare services, which makes these services more affordable to refugee patients with low socio-economic status, such as unemployed refugees with larger families [20]. Furthermore, it is indicated that refugees in the Azraq camp fared worse than those in the Al-Za’atari camp. Possible explanations for this outcome could be that refugees in the Azraq camp tend to have less living space, severely poor health infrastructure, have half the number of household assets as refugees in Al-Za’atari on average, and are less satisfied with services. However, refugees in Al-Za’atari may have better social networks and be in a better financial position. Further research is needed to better understand these differences.

This study disclosed that Syrian refugees in Jordan suffer from moderate to high levels of food insecurity. Food insecurity is a universal dilemma that faces migrants and refugees [21]. In terms of economic status, high-income countries (HIC) have received over half of the entire refugees in the world, while a significant percentage of them are accommodated in low- or middle-income countries [22,23]. Although the refugees have settled in HIC, the literature showed they still suffer from food insecurity. Comparatively, the food insecurity dilemma becomes more significant among those who settled in low- or middle-income countries [23]. Many reasons could be behind food insecurity, such as language obstacles, cultural reasons, maladaptation, and lack of financial support [24,25]. It has been found that, in low- or middle-income countries, such as Jordan, lack of financial support is considered the most influential reason behind food insecurity [22]. The findings of the present study are parallel with what has been found in the literature [26]. It appears that the reasons with the highest agreement rate were “have been worried that food would run out before you got money to buy more” and “the food that was bought just didn’t last, and there wasn’t any money to get more”. In addition, among the NCDs, the most common diagnoses reported were diabetes mellitus and hypertension. Their reported prevalence rose rapidly after 30 years of age. The observed pattern of refugees not seeking care because of not understanding their disease was congruent with a study carried out by Levindo de Siqueira and colleagues [27].

### 4.1. Implications

The current study provides clear information for officials, health administrators, policymakers, and researchers for developing a holistic strategy to facilitate Syrian refugees’ integration and support a range of goals in healthcare. Initiatives to improve the living conditions of socially disadvantaged refugees, through taking effective multidimensional policy steps that ensure they get fair treatment and respect for their essential rights, are critical. Moreover, appropriate steps must be taken to guarantee that refugees are treated equally in terms of removing healthcare accessibility difficulties, facilitating healthcare utilization, and improving meeting of their healthcare needs, by strengthening the primary healthcare services by providing mobile healthcare clinics that are easily connected to the effective free secondary and tertiary healthcare system. Furthermore, actions must be taken to increase their health insurance coverage.

### 4.2. Strengths and Limitations

The present study is considered the first in Jordan to address the Syrian refugees’ health status and looked at a variety of factors that could influence refugees with chronic diseases’ ability to access, as well as utilize, healthcare services. These findings may help researchers better determine which areas of healthcare systems or initiatives should be prioritized to enhance refugees’ equitable healthcare services. Moreover, most constructs were described using unique psychometric scales with proven reliability and validity in diverse populations, which is a particular strength of this research. This study also used a powerful instrument tool that was used for long time and proved its accuracy. Furthermore, the study sample was taken from two different camps, which enhances the representativeness and generalizability of the results. The findings of this study can be used as basic information for officials, legislators, and decision-makers to draw on.

There are several limitations in this study. The first is that it was designed with a cross-sectional technique. The second is that the study approach was a self-reported sample, which may have resulted in recall bias and affected the survey results’ accuracy.

### 4.3. Recommendations for Further Studies

This study provides useful findings regarding the social determinants of access to healthcare, as an indicator of health equity among Syrian refugees with chronic diseases. In addition, this study highlights the barriers that patients faced in accessing healthcare services. Therefore, it forms baseline data for future local studies. However, it is recommended that future national health surveys should be conducted to measure the Syrian refugees’ health, characteristics, accessibility to healthcare services and related barriers, and experiences of and satisfaction with healthcare provided. Furthermore, a larger survey regarding access to the public healthcare system for both refugees is critical. Moreover, a comparative study of healthcare access for Syrian refugees versus Jordanians would also be beneficial for gaining clarity on the scope of this issue, as well as help direct appropriate interventions to the populations most in need. Furthermore, it will also strengthen the research findings if future research included a qualitative approach that explores patients with other health problems.

### 4.4. Generalizability

Due to the importance of equitable access to healthcare services in improving health status among not only patients with chronic diseases but also the general population, there is an increasing need to emphasize the importance of sociodemographic determinants of accessing health and their impact on equity. Consequently, the findings of this study revealed that there are gaps in how participants utilize and access healthcare services, which contributes to differences in actual and perceived health status. Lastly, the study showed that the existence of these gaps could aid health officials, decision-makers, and researchers to boost and improve healthcare access among Syrian refugees.

## 5. Conclusions

It is concluded, therefore, that important gaps exist in terms of the utilization of healthcare among Syrian refugees. The reasons behind this include a lack of preparedness in the healthcare system to respond to the surge of patients and the added cost of care, despite the fact that the Jordanian government has stepped up and provided a reliable system to deal with the refugees’ healthcare needs because of the huge number of refugees from the Syrian crisis. Therefore, both decision-makers and healthcare managers need to make more efforts to improve accessibility and utilization of healthcare services for refugee communities.

## Figures and Tables

**Table 1:** Demographic Characteristics.

Socio-Demographics Characteristics Mean (SD)	n	%
Age in years (49.45, SD = 10.48) Gender		
Male	181	39.8
Female	274	60.2
Marital Status		
Single	9	2
Married	290	63.7
Divorced	19	2.9
Widowed	143	31.4
Resident home		
Al-Za’atari	300	65.9
Azraq	155	34.1
Family size		
<5	137	30.1
5–8	300	65.9
>9	18	4
Educational level		
Illiterate	117	25.7
Elementary	230	50.5
Below high school	78	17.1
High school	19	4.2
Diploma	7	1.5
Employment Status		
Not working	379	83.3
Own business	8	1.8
Employee	68	14.9
Monthly income in JD (mean = 42.37, SD = 73.07)		

**Table 2:** General Health Characteristics (N = 455).

Health Behavior/General Health (N = 455)	n	%
Five most chronic diseases		
Hypertension	108	23.7
Diabetes mellitus	119	26.2
Cardiovascular diseases	99	21.7
Chronic respiratory diseases	70	15.4
Arthritis	59	13
Smoking (cigarettes)		
Yes	102	22.4
No	353	77.6
Smoking (shisha)		
Yes	12	2.6
No	443	97.4
Health insurance?		
Yes	5	1.1
No	450	98.9
Frequency of follow-up (visit per month)		
Not regular	4	0.9
Once	349	76.7
Twice	55	12.1
Three times	16	3.5
Four times	31	6.8

**Table 3:** Indicators of the Accessibility Difficulties to Healthcare Services.

Predictors	Unstandardized Coefficients		Standardized Coefficients	t	Sig.	95% Confidence Interval for B	
	B	Std. Error	Beta			Lower Bound	Upper Bound
(Constant)	24.758	11.509		2.151	0.032	2.139	47.378
Age	−0.011	0.058	−0.009	−0.197	0.844	−0.126	0.103
Gender	0.193	1.031	0.007	0.187	0.852	−1.833	2.219
Social status	−0.074	0.662	−0.005	−0.111	0.912	−1.375	1.228
Family number	−0.830	1.121	−0.028	−0.740	0.459	−3.033	1.373
Living place	14.106	1.347	0.494	10.475	0.000	11.460	16.753
Working status	−1.362	0.778	−0.072	−1.751	0.081	−2.892	0.167
Insurance	6.367	4.608	0.049	1.382	0.168	−2.688	15.423
Self-perception of stress in life	−0.395	0.599	−0.027	−0.659	0.510	−1.572	0.782
Self-perception of physical health	0.630	0.974	0.034	0.648	0.518	−1.283	2.544
Self-perception of mental health	−1.036	1.030	−0.053	−1.006	0.315	−3.060	0.987
Satisfaction with healthcare services	−0.286	0.605	−0.021	−0.472	0.637	−1.475	0.904
Food insecurity	0.320	0.154	0.082	2.078	0.038	0.017	0.624
Perceived distance to healthcare services	3.039	0.739	0.147	4.110	0.000	1.586	4.492
Satisfaction with the waiting time to get healthcare services	−2.626	0.891	−0.137	−2.947	0.003	−4.378	−0.875

**Table 4:** Indicators of the unmet healthcare needs.

Predictors	Unstandardized Coefficients		Standardized Coefficients	t	Sig.	95% Confidence Interval for B	
	B	Std. Error	Beta			Lower Bound	Upper Bound
(Constant)	22.580	7.068		3.194	0.002	8.687	36.472
Age	0.021	0.036	0.024	0.588	0.557	−0.049	0.091
Gender	−1.572	0.633	−0.086	−2.483	0.013	−2.816	−0.328
Social status	0.282	0.407	0.030	0.692	0.489	−0.518	1.081
Family number	2.199	0.688	0.112	3.194	0.002	0.846	3.551
Living place	5.020	0.827	0.265	6.070	0.000	3.395	6.646
Working status	−1.721	0.478	−0.138	−3.602	0.000	−2.661	−0.782
Insurance	0.036	2.830	0.000	0.013	0.990	−5.526	5.598
Self-perception of stress in life	−0.569	0.368	−0.059	−1.546	0.123	−1.292	0.154
Self-perception of physical health	−3.431	0.598	−0.280	−5.738	0.000	−4.607	−2.256
Self-perception of mental health	−0.486	0.632	−0.038	−0.768	0.443	−1.729	0.757
Satisfaction with healthcare services	−0.701	0.372	−0.076	−1.885	0.060	−1.431	0.030
food insecurity	0.418	0.095	0.161	4.414	0.000	0.232	0.604
Perceived distance to healthcare services	−0.226	0.454	−0.016	−0.498	0.618	−1.119	0.666
Satisfaction with the waiting time to get healthcare services	−1.052	0.547	−0.083	−1.921	0.055	−2.127	0.024

## Data Availability

Not applicable.

## References

[1] United Nations Human Rights UNHCR Continues to Support Refugees in Jordan throughout 2019. https://www.unhcr.org/jo/12449-unhcr-continues-to-support-refugees-in-jordan-throughout-2019.html.

[2] Sida & UNHCR (2018). Assessing the Needs of Refugees for Financial and Non-Financial Services—Jordan. https://reliefweb.int/report/jordan/final-report-assessing-needs-refugees-financial-and-non-financial-services-jordan-13.

[3] International Medical Corps (2020). Utilization of Mental Health and Psychosocial Support Services among Syrian Refugees and Jordanians. https://reliefweb.int/report/jordan/utilization-mental-health-and-psychosocial-support-services-among-syrian-refugees-and.

[4] WHO (2017). Health is a Fundamental Human Right. https://www.who.int/news-room/commentaries/detail/health-is-a-fundamental-human-right/.

[5] WHO (2020). Human Rights and Health. https://www.who.int/news-room/fact-sheets/detail/human-rights-and-health.

[6] Venema H.U., Garretsen H.F., Van Der Maas P.J. (1995). Health of Migrants and Migrant Health Policy, The Netherlands as An Example. Soc. Sci. Med..

[7] WHO (2022). Noncommunicable Diseases: Mortality. https://www.who.int/data/gho/data/themes/topics/topic-details/GHO/ncd-mortality.

[8] Alwan A. (2010). Global Status Report on Noncommunicable Diseases 2010.

[9] Strong K., Wald N., Miller A., Alwan A.W., WHO Consultation Group (2005). Current Concepts in Screening for Noncommunicable Disease: World Health Organization Consultation Group Report on Methodology of Noncommunicable Disease Screening. J. Med. Screen..

[10] Van Minh H., Xuan Tran B. (2012). Assessing the Household Financial Burden Associated with the Chronic Non-Communicable Diseases in a Rural District of Vietnam. Glob. Health Action.

[11] Kien V.D., Van Minh H., Giang K.B., Dao A., Tuan L.T., Ng N. (2016). Socioeconomic Inequalities in Catastrophic Health Expenditure and Impoverishment Associated With Non-Communicable Diseases in Urban Hanoi, Vietnam. Int. J. Equity Health.

[12] Alduraidi H., Hamaideh S.H., Hamdan-Mansour A. (2021). Determinants of Personal Social Capital among Syrian Refugees: Comparison of Inside and Outside Camps Residence. Ment. Health Soc. Incl..

[13] Rehr M., Shoaib M., Ellithy S., Okour S., Ariti C., Ait-Bouziad I., van den Bosch P., Deprade A., Altarawneh M., Shafei A. (2018). Prevalence of Non-Communicable Diseases and Access to Care among Non-Camp Syrian Refugees in Northern Jordan. Confl. Health.

[14] Doocy S., Lyles E., Hanquart B., Woodman M. (2016). Prevalence, Care-Seeking, and Health Service Utilization for Non-Communicable Diseases among Syrian Refugees and Host Communities in Lebanon. Confl. Health.

[15] Al-Rousan T., Schwabkey Z., Jirmanus L., Nelson B.D. (2018). Health Needs and Priorities of Syrian Refugees in Camps and Urban Settings in Jordan: Perspectives of Refugees and Health Care Providers. East. Mediterr. Health J..

[16] Doocy S., Lyles E., Roberton T., Akhu-Zaheya L., Oweis A., Burnham G. (2015). Prevalence and Care-Seeking for Chronic Diseases among Syrian Refugees in Jordan. BMC Public Health.

[17] Maconick L., Ansbro É., Ellithy S., Jobanputra K., Tarawneh M., Roberts B. (2020). “To Die Is Better For Me”, Social Suffering Among Syrian Refugees at a Noncommunicable Disease Clinic in Jordan: A Qualitative Study. Confl. Health.

[18] Ratnayake R., Rawashdeh F., AbuAlRub R., Al-Ali N., Fawad M., Hani M.B., Greenough P.G., Al-Amire K., AlMaaitah R., Parmar P. (2020). Access to Care and Prevalence of Hypertension and Diabetes Among Syrian Refugees in Northern Jordan. JAMA Netw. Open.

[19] Alduraidi H., Waters C.M. (2017). Health-Related Quality of Life of Palestinian Refugees Inside and Outside Camps in Jordan. Nurs. Outlook.

[20] McNatt Z.Z., Freels P.E., Chandler H., Fawad M., Qarmout S., Al-Oraibi A.S., Boothby N. (2019). “What’s Happening in Syria Even Affects The Rocks”: A Qualitative Study of the Syrian Refugee Experience Accessing Noncommunicable Disease Services in Jordan. Confl. Health.

[21] Department of Economic and Social Affairs, United Nations, New York 2018. ST/ESA/SER.A/404. https://www.un.org/en/development/desa/population/migration/publications/migrationreport/docs/MigrationReport2017_Highlights.pdf.

[22] International Migration 2019: Wall Chart. https://www.un.org/en/development/desa/population/migration/publications/wallchart/docs/MigrationStock2019_Wallchart.pdf.

[23] Pakravan-Charvadeh M.R., Vatanparast H., Flora C. (2021). Food Insecurity Status of Afghan Refugees is Linked to Socioeconomic and Resettlement Status, Gender Disparities and Children’s Health Outcomes in Iran. Child Indic. Res..

[24] Lawlis T., Islam W., Upton P. (2018). Achieving the Four Dimensions of Food Security for Resettled Refugees in Australia: A Systematic Review. Nutr. Diet..

[25] Zuntz A.C., Klema M., Abdullateef S., Mazeri S., Alnabolsi S.F., Alfadel A., Boden L. (2022). Syrian Refugee Labour and Food Insecurity in Middle Eastern Agriculture during The Early COVID-19 Pandemic. Int. Labour Rev..

[26] Theofanidis D., Karavasileiadou S., Almegewly W. (2022). Post-traumatic stress disorder among Syrian refugees in Greece. Front. Psychiatr..

[27] Levindo de Siqueira I., Guimaraes R., Pagotto V., Rosso C., Batista S., Barbosa M. (2022). Access and Use of Health Services by People with Diabetes from the Item Response Theory. Int. J. Environ. Res. Public Health.

